# Mechanism of Acupuncture and Moxibustion on Chronic Prostatitis/Chronic Pelvic Pain Syndrome: A Narrative Review of Animal Studies

**DOI:** 10.1155/2021/2678242

**Published:** 2021-12-08

**Authors:** Xiaoling Wu, Kai Cheng, Chang Xu, Shaoming Liu, Qianhui Sun, Zhiwen Yang, Xingye Dai, Na Li

**Affiliations:** ^1^College of Acupuncture-Moxibustion and Tuina, Beijing University of Chinese Medicine, Beijing 100029, China; ^2^Dongfang Hospital Affiliated to Beijing University of Chinese Medicine, Beijing 100078, China

## Abstract

Chronic prostatitis/chronic pelvic pain syndrome (CP/CPPS) is a genitourinary disease commonly seen in males, with symptoms involving pelvic pain, urinary system disease, and sexual dysfunction, which seriously affects physical and mental health, and it also influences the quality of life of patients. At present, the disease's aetiology and pathogenesis are unclear, and there is also no effective treatment for it. Acupuncture and moxibustion have been a way to CP/CPPS, showing good curative effect with advantages of safety and affordability. However, the relevant research in this field is less discussed. By adopting databases, such as CNKI, VIP, Wanfang, PubMed, and Medline, this review article used keywords including chronic prostatitis, chronic pelvic pain syndrome, and electric acupuncture, manual acupuncture, moxibustion, and animal experiments, rats, mice, and mechanism research and reviewing research papers published from 1998 to 2021. Then, it further summarized and evaluated the mechanism research and gave a brief comment about modeling methods, acupoints selection, and stimulus parameters that have been used in the selected research papers. Equally important, this review article proposes a reference for the in-depth study of the mechanism of acupuncture and moxibustion on CP/CPPS and provides a theoretical basis to better treat the disease in the clinic.

## 1. Introduction

Chronic prostatitis/chronic pelvic pain syndrome (CP/CPPS) is a genitourinary disease that frequently occurs in adult males accounting for 10%–15% of males [1]. The typical clinical symptoms of CP/CPPS include chronic pain in the lower abdomen, perineum, and abnormal urination. Severe conditions can also cause sexual dysfunction. The clinical features of CP/CPPS are lingering and challenging to heal and recurrent attacks. This seriously affects physical and mental health of patients and leads to financial burdens to patients [2]. The aetiology of CP/CPPS remains unclear, and hypotheses about its pathogenesis are diverse, involving immune response, cytokines, microcirculation, oxidative stress, trace elements, nerves, endocrine system, adhesion molecules, and energy theory [3]. In CP/CPPS treatment, Western medicine primarily uses anti-inflammatory drugs, nonsteroidal anti-inflammatory and analgesic drugs, alpha-receptor antagonists, and other drugs. However, this drug treatment has disadvantages, such as high adverse reaction rate, easy drug resistance, and easy recurrence [4]. In recent years, scholars have developed a specific phenotypic classification system “UPOINT” based on patients' different causes, symptoms, and course [5]. According to their condition, the system can classify patients into more phenotypes and formulate a comprehensive treatment plan with individual specificity. However, its effectiveness and practicality need to be further verified [6].

In Chinese medicine treatment, CP/CPPS is generally used clinically by herbs, acupuncture, and moxibustion. As a proven and relatively safe alternative to nondrug treatments, acupuncture and moxibustion have been widely used in clinical practice. It has the advantages of simple operation, safety, and low treatment cost [7].

Acupuncture and moxibustion are a form of alternative medicine and a key component of traditional Chinese medicine that can be traced back to at least 2000 years [8]. Acupuncture is the insertion and stimulation of needles at specific points to promote the recovery of health. There are various methods of acupuncture, such as electric acupuncture (EA) and manual acupuncture (MA). Recent clinical studies have shown that acupuncture promotes efficacy for patients with CP/CPPS relieving pain, urinary symptoms and improving the QOL and NIH-CPSI total score, and the efficacy of acupuncture on CP/CPPS, including anti-inflammatory, neural, and immune modulation [9].

Moxibustion delivers heat stimulation at acupoints on the body surface by burning moxa leaves or moxa floss. Several animal and human studies have proved the analgesic and anti-inflammatory effects of moxibustion for CP/CPPS. The heat stimulation of moxibustion could improve the microcirculation and metabolism of the prostate, thereby eliminating chronic congestion and swelling by dilating the blood vessels and improving blood flow. A recent study [10] has shown that acupuncture treatments differ greatly from moxibustion in CP/CPPS treatment. The treatment mechanism of moxibustion for CP/CPPS mainly depends on heat stimulation of vessels and tissues. At the same time, acupuncture achieves analgesia and detumescence in CP/CPPS through needling stimulation of postganglionic sympathetic fibers, improving the release of catecholamines and endogenous opioid peptides.

**Figure 1:**
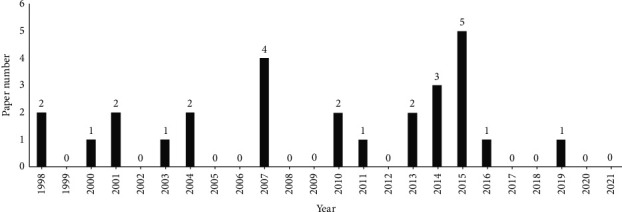
Annual distribution of papers on animal experimental studies on mechanism of acupuncture and moxibustion on CP/CPPS.

Although the efficacy of acupuncture and moxibustion on CP/CPPS has been affirmed in clinical practice, the mechanism of acupuncture and moxibustion for CP/CPPS has not been widely studied and elucidated. In particular, the mechanism of acupuncture and moxibustion on CP/CPPS animal models is still not enough. This article thus summarizes research articles published between 1998 and 2021, through keywords, including chronic prostatitis, chronic pelvic pain syndrome, electric acupuncture, manual acupuncture, moxibustion, acupoint embedding and animal experiments, rats, mice, and mechanism research. The databases include CNKI, VIP, Wanfang, PubMed, and Medline. There are a total of 27 retrieved research articles (see Figure 1). This paper provides a reference for the in-depth study of the mechanism of acupuncture and moxibustion on CP/CPPS and offers a theoretical basis for acupuncture and moxibustion to better prevent and treat the disease in the clinic.

## 2. Acupoints Selection

**Figure 2:**
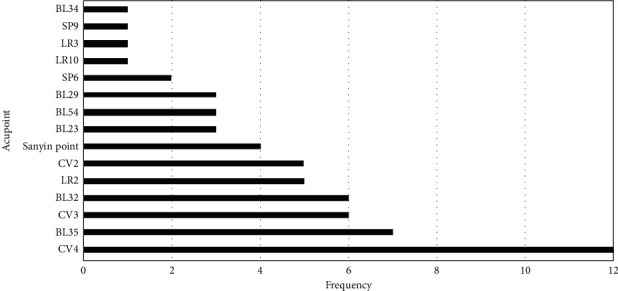
The frequent acupoints and their frequencies on animal studies. The *y*-axis indicates the acupoints used. The *x*-axis indicates the frequencies of acupoint used on animal experimental studies from 1998 to 2021.

**Figure 3:**
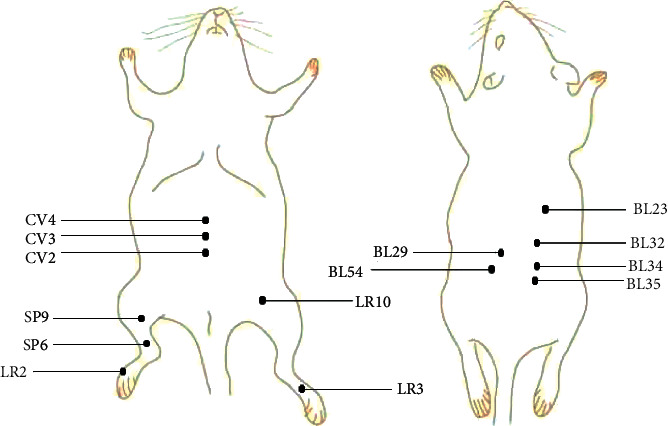
The location of frequent acupoints in rat in animal studies.

Acupoints are the unique parts of the human viscera, meridians, and collaterals for the infusion of qi and blood. They are the reaction points of diseases and the stimulation points of treatment. The current research shows that the acupuncture and moxibustion on CP/CPPS have different points in related animal experiments. Through observation and summary, we summarized the frequency of commonly used acupoints (see Figure 2). The following acupuncture points are more likely to be used on CP/CPPS animal models by researchers: Guanyuan (CV4), Huiyang (BL35), Zhongji (CV3), Ciliao (BL32), Xingjian (LR2), Qugu (CV2), Sanyin points (Jiayin 1, Jiayin 2, and Chong yin), Shenshu (BL23), Zhibian (BL54), and Sanyinjiao (SP6). By referring to the rat acupoint positioning recorded in [11], we drew a schematic location of acupoints mentioned above, as shown in Figure 3.

## 3. The Mechanism of Acupuncture and Moxibustion on CP/CPPS

### 3.1. The Holistic Regulation of Acupuncture and Moxibustion

#### 3.1.1. Acupuncture Regulates the Immune System

In modern medicine, the local inflammatory response of CP/CPPS is closely related to its autoimmune response. In the CP/CPPS mouse model studied by Miao et al. [12] under the light microscope, the thymus cortex, which is closely related to immune response, was significantly thinned and lymphocytes decreased, suggesting that CP/CPPS is related to the decline of autoimmune function. Many other studies have shown that the therapeutic effect of acupuncture on CP/CPPS animal models is closely related to the regulation of the inflammatory response and immune response during the development of CP/CPPS. For example, Liu et al. [13] found that the serum CD4^+^ and CD4^+^/CD8^+^ ratio of CP/CPPS rats prepared by intradermal injection of complete Freund's adjuvant (CFA) combined with prostaglandin purification solution were significantly reduced. The ratio of CD4^+^/CD8^+^ is positively correlated with the degree of inflammation in the body. Excessive immunity caused by long-term inflammation will make the body's CD4^+^, CD8^+^ content, and CD4^+^/CD8^+^ ratio imbalance [14]. After EA at Sanyin points, frequency 2 Hz/100 Hz, voltage 24 V, CD4+ and CD4+/CD8+ ratio increased. It is suggested that acupuncture can exert an effect on CP/CPPS by promoting the homeostasis of the immune system of CP/CPPS rats and the recovery of overall immune function. IL-2, produced by activated T lymphocytes, contains high levels in the prostatic fluid (EPS) of CP/CPPS patients. The main biological activity of IL-2 is to promote the production of other lymphocytes and antibodies, and it plays an important role in the body's immune response and regulation. Researches by Zhao et al. [15], Yan et al. [16], Hui et al. [17], and others all suggest that acupuncture can reduce the expression of IL-2 in the CP/CPPS rat model to exert its treatment effect. In addition, IgG, as the main body's anti-infection antibody, has an immune regulation effect. A study [18] found that IgG in the prostate tissue homogenate of CP/CPPS rats was at a high level. For acupuncture at Sanyin points, the specific operation is MA at 1 cm to Jiayin 1 and Jiayin 2 points and at 1.5 cm to Chongyin points, with flattening, reinforcing-reducing method, keeping the needle for 20 minutes, once a day, 15 times. The results suggest that acupuncture can reduce the IgG of CP/CPPS rats, improve local humoral immune response, and improve inflammation. The study by Yi et al. [19] found that phagocytosis of leukocytes function and T cell count in the serum of CP/CPPS rats were significantly lower than those in normal rats. This suggests that the overall nonspecific immune function is also an important factor in the development of CP/CPPS. Acupuncture can indeed restore and maintain the low immune function of the CP/CPPS rat model to a normal level in a short time. It can be seen that acupuncture improves the body's immunity and enhances anti-inflammatory ability, which is one of the important mechanisms for acupuncture to obtain effect.

#### 3.1.2. Acupuncture Regulates Endocrine

The imbalance of the endocrine has a specific relationship with the pathogenesis of CP/CPPS. In particular, changes in hormone levels are significantly related to the occurrence of CP/CPPS. Shinsuke et al. [20] observed decreased estrogen receptor expression in a CP/CPPS model with increased inflammation-related gene expression. However, estrogen receptor agonists can inhibit prostate inflammation. The results of Jia et al. [21] and other studies suggest that taking testosterone (T) can inhibit the prostate inflammation in CP/CPPS rat model made by subcutaneous injection of estradiol after Castration. T can regulate the related functions of the prostate through various ways and plays an important role in the normal development and maintenance of the prostate's normal function. As early as 1977, a study [22] found that the symptoms of CP/CPPS patients were closely related to the decrease of T secretion. Recent studies [23] also found that the serum T level decreased in the CP/CPPS rat model, and the heat-getting needle at BL32 can alleviate the inflammatory response by increasing the T level. Another study [19] found that the prostate contains vascular endothelin (ET). ET is the strongest known vasoconstrictor peptide, which can cause the smooth muscle contracts strongly. When patients with prostatic pain have received urodynamic examination, it was found that the bladder neck and prostatic urethrospasm occurred frequently. This may be related to the increase in ET content. The study by Zhang et al. [23] also found that the heat-getting needle at BL32 can reduce the excessively high ET in the prostate of CP/CPPS rats to normal levels and relieve their urinary tract related symptoms. This suggests that acupuncture can treat CP/CPPS by regulating the body's endocrine function.

### 3.2. The Partial Improvement of Acupuncture and Moxibustion

#### 3.2.1. Acupuncture Protects Damaged Prostate Tissue Structure

Through animal experiments, the prostate's microscopic and ultramicro histological structure can be observed under light and electronic microscopes [24]. Under a light microscope, a normal prostate gland can be seen to be hollow and rich in pink secretions. Under the electronic microscope, there are abundant rough endoplasmic reticulum and Golgi complex in the cytoplasm of acinar cells, and there is prostatic concretion in the glandular cavity. A large number of animal experiments have shown that CP/CPPS can cause prostatitis pathological changes. For example, Miao et al. [12] observed that the prostate tissue of CP/CPPS rat model prepared by Xiaozhiling injections into the prostate had obvious inflammatory cell infiltration. Other studies have shown that acupuncture can restore the physiological functions of the prostate by improving the pathological changes of the prostate tissue and protecting the ultrastructure of the prostate tissue. For example, Yi et al. [25] used a CP/CPPS model replicated by injecting carrageenan into the prostate of rats. The BL54 was taken with the needle pointed at the prostate area, 0.5–0.8 inches deep, and the twirling reinforcing-reducing method was performed, once a day ,10 min each time. After 9 consecutive days, under the light microscope, the prostatitis was found to be reduced, and under the electron microscope, the performance of the pathological damage of the prostate was effectively reversed. This result suggests that acupuncture can promote the metabolism of prostate tissue and enhance the prostate's secretory function and repair ability. Zhang et al. [26] studied the histology of CP/CPPS rats by heat-getting needle, using a 1-inch needle to imitate the downward force applied to the human body as a characteristic of the heat-getting needle, selecting BL32 for 10 minutes each time. After 12 days, the experimental results showed that acupuncture can accelerate the repair of the micro and ultrastructure of the diseased tissue. Liu et al. [27] took BL23 and BL35 on the CP/CPPS rat model, applied EA, stimulated with 2 Hz/100 Hz sparse-dense waves, 2 V voltage, and retained the needle for 30 minutes, once a day, 5 times as a course of treatment. After 4 courses of treatment, the results showed that the congestion and edema of prostatic stroma were alleviated, and the inflammatory cell infiltration was significantly subsided. It further shows that EA can improve the pathological changes of prostate tissue by affecting the process of tissue exudation, hyperplasia, and deterioration.

#### 3.2.2. Acupuncture and Moxibustion Regulate Cytokines in Prostate

Cytokines are mainly synthesized and secreted by immune cells. When the body has an inflammatory stress response, immune cells are activated to form a response, causing an inflammatory response. Cytokines are mainly divided into proinflammatory cytokines, anti-inflammatory cytokines, and regulatory cytokines [28]. Some studies believe that prostatitis is a chain reaction mediated by cytokines. Acupuncture can treat CP/CPPS by regulating cytokines [17]. For example, Hu et al. [29] found that the levels of IL-1*β* and TNF-*α* in the prostate tissue of the CP/CPPS rat model were significantly increased. At CV4, CV3, BL32, and Xialiao point (BL34), shenhualeihuo moxibustion was used for moxibustion once a day for 10 min. After 4 weeks of continuous moxibustion, the levels of IL-1*β* and TNF-*α* in the prostate tissue were significantly reduced. This suggests that acupuncture can reduce the level of proinflammatory cytokines, inhibit the inflammatory response, and achieve the purpose of treating CP/CPPS. As an anti-inflammatory cytokine, IL-10 can inhibit the inflammatory response and maintain the balance of the inflammatory factor network by inhibiting the production of various proinflammatory factors such as TNF-*α*, IL-1*β*, and IL-8. Zhu et al. [30] found that the IL-10 content in the prostate homogenate of CP/CPPS rat model decreased, and EA at CV4, CV3, Zuwuli (LR10), Yinlingquan (SP9), and Taichong (LR3). Using continuous wave, once every other day, after a total of 15 treatments, IL-10 increased. At the same time, it was also found that the proinflammatory factors TNF-*α*, IL-6, and IL-8 in prostate tissue homogenate were significantly reduced. This suggests that acupuncture can treat CP/CPPS by regulating proinflammatory cytokines and anti-inflammatory cytokines. At the same time, it is suggested that acupuncture can simultaneously adjust the complex network system composed of multiple cytokines and restore the balance of the immune state that has been imbalanced. This improves the local inflammation of the prostate, promotes the repair of the prostate tissue, and protects the prostate tissue.

#### 3.2.3. Acupuncture Regulates Pain-Related Factors in Prostate

One of the typical clinical symptoms of CPCPPS is a pain in the lower abdomen and perineum. Ilhan et al. [31] found that in the experiment of analgesic effect of acupuncture on CP/CPPS rats, the pain threshold of model rats was significantly reduced. Acupuncture at CV4, CV3, BL32, and BL35 was performed one hour at a time and every 10 minutes. After 6 weeks, it was found that the pain threshold of rats was significantly increased. This suggests that acupuncture has a better analgesic effect on CP/CPPS. Studies have shown that the cause of the pain may be the inflammatory response caused by various factors that causes prostate tissue damage, which causes the prostate tissue to release some pain substances, such as *β*-endorphin (*β*-EP), cyclooxygenase-2 (COX-2), and prostaglandin E2 (PGE2). These pain substances indirectly increase the excitability of nociceptors and sensitization of nociceptors by changing the endobiochemical microenvironment of the prostate [32,33]. Many studies have shown that the analgesic effect of acupuncture on CP/CPPS is closely related to the regulation of pain substances. Yan [34] and others found that COX-2 was strongly positively expressed in the prostate of CP/CPPS rats. After EA at Sanyin points, the expression of COX-2 decreased, and the pain threshold of rats increased. This suggests that acupuncture can relieve pain by reducing COX-2 expression. In the study of Liu et al. [27] and others, it was found that after EA at CV4, CV3, LR10, SP9, and LR3, the *β*-EP of model rats increased. This suggests that the analgesic effect of acupuncture on the CP/CPPS model can be achieved by increasing *β*-EP. The strong positive expression of COX-2 can also cause a large amount of PGE2 synthesis in prostate tissue, and PGE2 is positively correlated with pain [35]. Yang et al. [36] performed EA treatment on the CP/CPPS rat model with BL35 and Zhong lvshu (BL29). The wave was 4 Hz, the dense wave was 20 Hz, the voltage was 4–6 V, and the needle was retained for 20 minutes, once a day for 10 days. The results of treatment found that the decreased PGE2 of CP/CPPS rats increased, suggesting that one of the mechanisms of acupuncture's analgesic effect on CP/CPPS rats is to promote the synthesis of PGE2.

#### 3.2.4. Acupuncture Increases Energy Production in Prostate

The energy balance of a healthy body is mainly reflected in the production, utilization, and heat production of ATP. Studies have found [37] that the activity of sodium-potassium-adenosine triphosphate (Na^+^-K^+^-ATPase) in CP/CPPS model rats is generally significantly reduced. After the heat-getting needle was applied to the BL32 in the rat, the low Na^+^-K^+^-ATPase activity in the prostate tissue of the CP/CPPS model rat returned to the normal level, the energy production was improved, and the disease was repaired. It can be seen that the effect of acupuncture on CP/CPPS may be related to energy regulation, and acupuncture promotes the generation and effect of body energy.

#### 3.2.5. Acupuncture Regulates Oxidative Stress in Prostate

In the long-term evolution of organisms, the organism's tissues and cells have established a complete antioxidant defence system to defend against free radicals' damage and destruction. In particular, total antioxidant capacity (T-AOC) is high, and the main product of oxygen free radicals is malondialdehyde (MDA). The change of its content can often reflect the degree of lipid peroxidation in the body and indirectly reflect the degree of cell damage [38]. A study [39] found that CP/CPPS rats were injected intracutaneously with CFA combined with prostaglandin purification solution, the T-AOC level in the prostate of the rat decreased, and the MDA level increased. This indicates that the prostate tissue's local antioxidant capacity is reduced, and the tissue is overoxidized, causing damage to the prostate tissue. EA at the Sanyin points at a frequency of 10 Hz and retaining the needle for 20 minutes, once a day for a total of 15 times, can increase the level of T-AOC and decrease the level of MDA. This suggests that acupuncture can enhance local tissues' antioxidant defence capabilities, protect prostate tissue structure, and reduce excessive oxidative damage to tissue cells.

#### 3.2.6. Acupuncture Regulates Microcirculation in Prostate

Microcirculation refers to the blood circulation between arterioles and venules. Microcirculation disturbances can lead to a hypercoagulable state as well as high coagulation and high adhesion state, which leads to severe consequences such as ischemia, hypoxia, and metabolic dysfunction [40]. Long-term chronic inflammation often leads to an increase in whole blood viscosity in the prostate, causing disturbance of blood circulation in the prostate. This result is consistent with Ju's research group's experimental results on the increase in the whole blood viscosity of CP/CPPS model rats at different shear rates [41]. The research results of the research group also showed that there is a certain positive correlation between changes in blood rheology and the severity of CP/CPPS. Acupuncture and moxibustion can relieve CP/CPPS pelvic pain and urinary dysfunction. Acupuncture can improve prostate blood circulation and eliminate local hyperemia and edema. Yi [42] and others observed that the blood supply to the prostate of the CP/CPPS rat model was significantly reduced, and the circulation volume was significantly decreased. Acupuncture at BL54 can speed up its blood flow and restore blood supply to the prostate. Combined with the histopathological results of early CP/CPPS animals, it was found that the pathological damage of CP/CPPS model after acupuncture was efficiently reversed [25]. This suggests that the mechanism of acupuncture on CP/CPPS may be related to acupuncture improving microcirculation, accelerating local blood flow, and improving tissue metabolism.

#### 3.2.7. Acupuncture Increases Zinc in Prostate

Zinc is an important trace element and immune defence factor in the human body [40], and it is also the trace element that has the greatest impact on the prostate. Zinc in prostate secretions and semen is higher than that in other organs and body fluids. High levels of prostate zinc are related to the anti-inflammatory ability of the prostate [43]. Zhang et al's research [44] showed that the serum zinc content of the CP/CPPS rat model was significantly reduced, after embedding thread therapy was applied to BL23, and CV4 a week for four weeks. This treatment can significantly increase the serum zinc content, indicating that serum zinc is a vital substance to maintain the prostate tissue structure and physiological function in a stable state. One of the mechanisms of acupoint catgut embedding in the treatment of CP/CPPS is related to increased serum zinc content.

#### 3.2.8. Acupuncture Regulates Adhesion Molecules in Prostate

Cell adhesion molecules are closely related to the regulation and mediation of various immune responses and tissue repair. In particular, the relationship between adhesion molecule-1 (ICAM-1) and CP/CPPS is incredibly close. ICAM-1-mediated cell adhesion is an essential basis for maintaining the body's normal defence system. However, the sustained or enhanced expression of ICAM-1 in tissue cells may cause inflammatory and immune damage in tissues and organs [45]. A study [46] found that the number of positive cells expressing ICAM-1 protein in rats' prostate tissue in the CP/CPPS model group was significantly increased. EA was carried out at the bilateral BL23 and BL35, once a day, five days as a course of treatment. After four treatment courses, it was found that acupuncture can significantly reduce ICAM-1 protein expression in prostate tissue. This suggests that one of the mechanisms that acupuncture inhibits prostate inflammation is achieved through the following process (i.e., reducing the expression of adhesion molecules such as ICAM-1 in the prostate tissue, inhibiting the adhesion of inflammatory cells, reducing the infiltration of inflammatory cells into the prostate tissue, and ultimately reducing the release of inflammatory mediators in the prostate tissue).

#### 3.2.9. Acupuncture Regulates Bladder Function

The typical clinical symptoms of CP/CPPS include frequent micturition, urgent micturition, and painful micturition. This is related to the inflammatory response of the prostate tissue and the hyperplasia and fibrosis of the tissue around the acinar, which further affects the systolic and diastolic function of bladder [47]. Studies [48] confirmed EA at BL35 and BL29, density wave 4-6V, needle retention for 20 minutes, once a day, a total of 7 times. It can lower high intravesical pressure, improve low compliance bladder, inhibit overactive bladder function, and increase urinary flow rate; it can also affect the process of tissue exudation, hyperplasia, and deterioration, thereby improving urodynamics [49]. Yang et al. [36] studied the effect of EA on the interstitial cell of Cajal (ICC) in the bladder of CP/CPPS rats from a more microscopic level. The study found that the number of ICC-like cells in the bladder detrusor muscle of CP/CPPS model rats increased significantly, the cell arrangement was disordered, and the connection between cells was interrupted. After the intervention of EA, the number of ICC-like cells decreased compared with the model group, the cells were arranged in an orderly manner, and the relationship between each other was significantly strengthened. This shows that EA can partially affect the ultrastructural changes of bladder tissue, thereby improving bladder function.

## 4. Summary of Animal Experiments on the Mechanism of Acupuncture and Moxibustion on CP/CPPS

In recent years, there have been various acupuncture methods in the animal experiment research of acupuncture and moxibustion treatment of CP/CPPS, including EA, MA, moxibustion, heat-getting needling warming-promotion needling, and acupoint catgut embedding. The intervention period of the experiment is mostly 7–42 days. However, there are certain differences in selecting acupuncture points and their combination, including anterior-posterior acupoints combination, distal-proximal acupoints combination, and points combination recorded in ancient medical books. CP/CPPS animal models and indicators are also rich and diverse. Given the special intervention methods and unique curative effects of acupuncture on CP/CPPS rats, this paper sorts out the relevant experimental details of the effects of acupuncture and moxibustion on CP/CPPS models obtained from the above retrieval, as shown in Table 1.

## 5. Discussion

The curative effect of acupuncture and moxibustion on CP/CPPS has been tested in practice and recognized by the academic community. Relevant animal experimental studies have reflected the holistic regulation and partial improvement of acupuncture and moxibustion, and it can promote tissue repair, improve pathological structure, regulate immune response, affect endocrine, adjust cytokine network structure, reduce urinary disorders, promote local microcirculation, and improve energy metabolism. However, there are still many shortcomings in animal experiments on the effect of acupuncture and moxibustion on CP/CPPS, and there are still some problems that need attention and discussion.

### 5.1. Animal Modeling Methods of CP/CPPS Related to Acupuncture and Moxibustion

**Figure 4:**
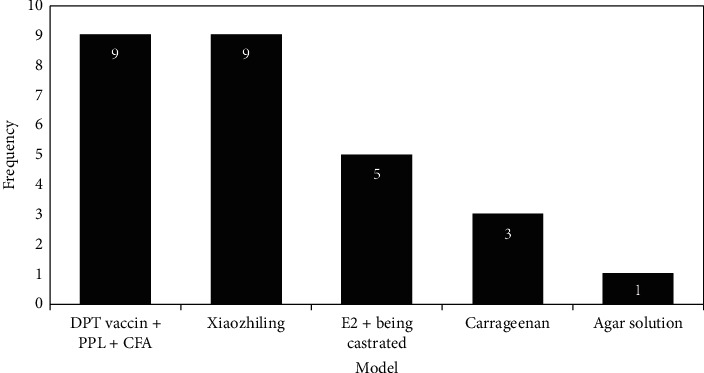
The frequent animal modeling methods and their frequencies on animal studies related to acupuncture and moxibustion. The *x*-axis indicates the animal modeling methods used; the *y*-axis indicates the frequencies of modeling methods used from 1998 to 2021. CFA: complete Freund's adjuvant; PPL: protein purification liquid; E2: estradiol; and DPT vaccine: diphtheria-pertussis-tetanus vaccination.

The first step to study the mechanism of the disease and explore new therapies is to establish a good CP/CPPS model. There are various CP/CPPS animal models currently, and their common feature is the appearance of nonbacterial inflammation of the prostate. In recent years, the following types of CP/CPPS models have been used in experimental studies on the effects of acupuncture on CP/CPPS: castration combined with estrogen induction, autoimmune modeling, direct injection of chemical agents, etc. (Table 1). By querying and summarizing relevant literature on the effects of acupuncture and moxibustion on CP/CPPS animal models in recent years, we find that CFA combined with prostaglandin intradermal injection was used 9 times, prostate internal Xiaozhiling injection was used 9 times, the rat castration combined with estrogen induction method was used 5 times, the intraprostatic carrageenan injection method was used 3 times, and the intraprostatic agar solution injection method was used once (see Figure 4). There are two reasons for the modeling: First, the chemical reagent injection modeling method is simple, short in a cycle, and reproducible [51]. For instance, a research team injected 0.2 ml of 25% “Xiaozhiling injection” into the dorsal lobe of the prostate and successfully created a CP/CPPS rat model after 7 days, which caused fibroproliferative inflammation of the prostate to be similar to the pathological changes of the prostate of patients with CP/CPPS [52]. Second, the intradermal injection of CFA combined with prostaglandin and rat castration combined with estrogen-induced modeling can cause local immune function in prostate tissue and disorder of animal hormone levels and damage prostate tissue cells. The modeling reagents of these two methods are easy to obtain and of low cost, high molding rate, and low mortality, and the model is stable and reliable and has good pathological specificity [53]. Therefore, the above methods are used more frequently and are more likely to be used as relevant animal models to study the mechanism of acupuncture on CP/CPPS. However, looking at the CP/CPPS models related to acupuncture and moxibustion, it can be seen that although there are various methods of animal modeling (see Figure 4), a recognized CP/CPPS animal model has not yet been prepared, and it lacks a certain degree of standardization. This may be related to the shortcomings of each modeling method. For example, the animal infection rate and mortality rate are higher after the chemical reagent injection modeling method [51]. Currently, the specific antigen protein that induces the immune response by the autoimmune modeling method remains unclear [54]. Regarding rat castration combined with estrogen-induced modeling, the modeling time is long, and the operation is cumbersome and complicated [55]. In addition, the CP/CPPS model has not yet corresponded to the main symptoms of CP/CPPS, and it is impossible to distinguish whether the CP/CPPS model is based on dysuria, pain, or simple prostate inflammation. Therefore, we should devote more efforts to the following research in the future: first, carrying out more animal model studies on the effect of acupuncture on CP/CPPS urination abnormalities and the analgesic effect of acupuncture on CP/CPPS; second, strictly standardizing the experimental design and striving to prepare a CP/CPPS animal model with symptomatic characteristics so that the experimental animal research of acupuncture intervention in CP/CPPS is closer to clinical reality.

### 5.2. Mechanism of CP/CPPS Model Related to Acupuncture and Moxibustion Research

**Figure 5:**
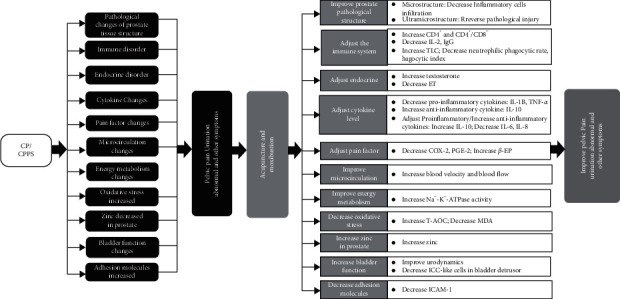
Summary of the proposed mechanisms by which acupuncture and moxibustion improves the symptoms in CP/CPPS.

Recent studies have shown that acupuncture can effectively improve the symptoms and pathological changes of CP/CPPS. Its mechanism of action is mainly related to the regulation of autoimmune response, endocrine, microcirculation, oxidative stress, cytokines, zinc ions, adhesion molecules, etc. (see Figure 5). At present, the mechanism research is still relatively weak because most of the CP/CPPS animal experiments related to acupuncture and moxibustion are mostly repeated at a low level, without in-depth and systematic exploration of possible signal pathways and targets, as well as the mechanism of acupuncture and moxibustion on CP/CPPS from the microscopic molecular aspects, such as proteins and genes. In addition, the mechanisms of the brain-related comorbidities of acupuncture and moxibustion for CP/CPPS are still largely unknown and lack experimental animal research. Recent studies have fully shown that the mechanism of brain-related comorbidities of CP/CPPS is closely related to the brain. Sutulovic et al. [56] showed that anxiety-like behavior in rats with CP/CPPS increased, and the potential mechanisms of observed behavioral alterations could result from an interplay between increased brain oxidative stress, elevated serum corticosterone level, and loss of hippocampal PV + interneurons. They [57] also showed that CP/CPPS increases susceptibility to lindane-induced seizures in rats associated with the increased level of IL-1*β* and IL-6 in the cortex and thalamus. Thus, the effect of acupuncture and moxibustion on brain-related symptoms in CP/CPPS and its mechanisms deserve further study. Therefore, it is necessary to expand further the research on the mechanism of acupuncture and moxibustion on CP/CPPS, integrating molecular biology, bioinformatics, epigenetics, and other multidisciplinary theories and further exploring the possible pathway targets of the mechanism of acupuncture and moxibustion on CP/CPPS.

### 5.3. Acupoint Selection of Acupuncture and Moxibustion Research

In acupoint selection, single acupoints or acupoint compatibility in the CP/CPPS animal experiments are rich and diverse, and there is no unified and standardized standard for acupoints and acupoint compatibility. *Lingshu Hailun* mentioned that there is a correlation between viscera and acupoints. However, most research has not been based on modern neuroanatomy to explore the correlation between acupuncture points and the prostate. Therefore, there is a lack of deep understanding of the relationship between the selected acupoint compatibility and CP/CPPS. Only a few studies do start from clinical experience and start to select acupoints based on discovering the relationship between acupoints and prostate from the perspective of modern neuroanatomy and to carry out acupoint compatibility. But some of these practices is the lack of supportable acupuncture meridian theory. Therefore, future research should integrate acupuncture theory with modern neuroanatomy and other disciplines. For example, through the use of big data technologies such as data mining technology and knowledge graphs, the issues of acupuncture and moxibustion treatment of CP/CPPS acupoints and their frequency of use and compatibility of acupoints are summarized, in order to excavate the internal laws of acupoint selection and acupoint compatibility, and finally, form a unified and standardized standard. At the same time, they are paying attention to the control of nonacupoints to verify the specific effects of acupoints fully.

### 5.4. Stimulation Parameters of Acupuncture and Moxibustion

In terms of acupuncture and moxibustion technology, this article discusses EA, MA, moxibustion, heat-getting needling, warming-promotion needling, and acupoint catgut embedding. However, the acupuncture and moxibustion stimulation parameters for the treatment of CP/CPPS lack standardization. In terms of MA, there is a lack of detailed elaboration on the measurement of acupuncture maneuvers such as the direction of the force, the magnitude of the force, the duration of the action, the interval between two times, and the depth of acupuncture. Moreover, this kind of acupuncture and moxibustion technique is easily affected by the subjective will of the researcher and is difficult to control and unstable. In terms of EA, although the amount of stimulation can be adjusted accurately, the EA stimulation parameters in many studies have not yet reached a unified standard. Different EA frequencies affect the body, and the neurotransmitters that can be released by stimulating the body are also different [58]. Therefore, it should be emphasized that acupuncture and moxibustion stimulation parameters of the effect of acupuncture on CP/CPPS should be combined with big data technology to establish a literature database for acupuncture treatment of CP/CPPS. The purpose is to explore the methods of acupuncture and moxibustion, the duration of action, the frequency of treatment, the length of treatment, etc., to formulate standard plans, compare the differences between the plans, and provide a basis for screening the best treatment plan.

## 6. Conclusion

In summary, this article examines the current research status of acupuncture and moxibustion on CP/CPPS animal models. In clinical trials, a multicenter, randomized, sham-controlled trial showed that 20 sessions of acupuncture over 8 weeks provided clinical relief of symptoms of moderate-to-severe CP/CPPS in a substantially higher proportion of participants. And the associated symptoms of pain, voiding dysfunction, anxiety, and depression also could be improved. Its efficacy may last 24 weeks after treatment, which is a long-term efficacy of acupuncture [59]. However, there is still no available evidence from sufficiently high-quality studies to draw a definite conclusion of the mechanisms of acupuncture and moxibustion in the treatment of CP/CPPS. Therefore, future research should make full use of modern advanced science and technology and research methods and strictly standardize experimental design, including standardization of models, acupoints, acupoint compatibility, and acupuncture stimulation parameters, to further explore the mechanism of the short-term and long-term efficacy of acupuncture and moxibustion on CP/CPPS, and to provide a more reliable theoretical basis for the clinical application of acupuncture and moxibustion.

## Figures and Tables

**Table 1:** Animal experimental research summary about the effect of acupuncture and moxibustion on CP/CPPS.

First/corresponding author	Animal/model/modeling time	Method	Acupoint	Stimulus parameters	Treatment course	Molecular and cellular results	Routine observation results
Hakan Akdere/İlhanÖztekin [31]	Male wistar rats; E2(0.25 mg/kg, sc) + DHT (0.25 mg/kg, sc);28 days	MA	CV3, CV4, BL32, BL35	Twisting the acupuncture needles at a frequency of once every 10 minutes. Then, the needles were retained for 60 min.	Three days a week, 6 weeks		Body weight↑
							Prostate weight↓
							Improve pain
							Improve
							prostate histopathology

Laixi Yi [19, 25, 42]	Male wistar rats; carrageenan (1%, 0.05 ml) injected into the prostate; 7 days	MA	BL54	The acupuncture needle points to the prostate area, depth 0.5–0.8 inches, twisting the acupuncture needles 10 min	Once a day 9 times	ET in prostate↓	Improve histopathology and ultrastructure of prostate the phagocytosis rate of neutrophils.Phagocytic index↓ the local blood flow velocity and blood flow of prostate↑

Xingke Yan/Tianyou He [34]	SPF male SD rats; DPT vaccine (0.5 ml，ip) ＋ protein purification liquid (15 mg/ml, sc) ＋ CFA (sc); 45 days	MA	Jiayin 1 Jiayin 2 Zhongyin	Twisting the acupuncture needles at a frequency of 1 minute and at an angle of 180 for 1 min. Then, the needles were retained for 20 minutes	Once a day 10 days	COX-2 in prostate and hypothalamus↓	Improve histopathology of prostate and hypothalamus

Xingke Yan/Tianyou He [13, 19]	SPF male SD rats; DPT vaccine (0.5 ml，ip) ＋ protein purification liquid (15 mg/ml，sc) ＋ CFA (sc); 45 days	EA	Jiayin1 Jiayin2 Zhongyin	10 HZ The intensity was measured by tail vibration 20 min	Once a day 15 days	CD4^+^/CD8 ^+^and CD4 + in plasma↑ IgG, IL-2, IL-8、INOS, TNF-*α*, MDA In prostate↓ T-AOC in prostate↑	Prostate weight and index↓

Bo Yang/Qinghui Qi [36]	Male SD rats; DPT vaccine (0.5 ml，ip) ＋ protein purification liquid (15 mg/ml, sc) ＋ CFA (sc); 45 days	EA	BL29 BL35	4Hz/20 Hz 4–6V 20 min	Once a day 10 days	IL-1, PGE2, *β*-EP in prostate↓ NGFmRNA in bladder detrusor is decreased↓	ICC cells IOD in bladder detrusor is increased↓

Wei Zhu/Qinghu He [30]	Male SD rats;E20.25 mg/kg(sc) after being castrated;30 days	EA	CV4, CV3, LR1, LR3, SP9	Continuous wave. The intensity was measured by tail vibration 20 min	Once every other day 15 times	TNF-*α*, IL-6, IL-8↓, *β*-EP, IL-10↑ in prostate	Improve prostate histopathology

Shilin Li [50]	Male wistar rats;E20.25 mg/kg(sc) after being castrated;30 days	MA	CV4, CV3, BL23, BL35, SP6	Twisting the acupuncture needles at a frequency of 100 times/min and at an angle of 180 for three min, twisting every 10 minutes, then, the needles were retained for 30 minutes	Once every day 5 days for a course 4 courses 2 days rest between course	IL-1*β*, TNF-*α*, MIF↓ in prostate	Improve prostate histopathology
Tingting Liu/Weibing Gao [27]	Male wistar rats; Xiaozhiling (25%, 0.2 ml) injected into the prostate; 14 days	MA or EA	EA：BL23 BL35 MA：SP6 CV4, CV3	EA：2 Hz/100 Hz、2V、30 min. MA：Twisting the acupuncture needles at a frequency of 100 times/min and at an angle of 90–120 for 3 min, twisting every 10 minutes, then, the needles were retained for 30 minutes.	Once every day 5 days for a course 4 courses 1 day rest between course	TNF-*α*、IL-2 in serum↓ ICAM-1 in prostate↓	Improve prostate histopathology

Yaodong Zhao [15, 32, 35]	Male wistar rats;Xiaozhiling (25%, 0.2 ml)injected into the prostate; 9 days	Warming-promotionneedling	CV4, CV2 LR2	The right thumb forward 9 times, then，heavy insertion and light lifting continuous 9 times the right thumb forward 9 times again, once every 15 min, 30 min	1 time every day 30 days	TNF-*α*, IL-*β*↓in serum IL-6, IL-2 in serum↓	

Baowen Zhang [23, 26, 37]	Male wistar rats;Xiaozhiling (25%, 0.2 ml)injected into the prostate;7 days	Heat-gettingneedling	BL32	1-inch filiform needle needing downword with force, then, the needles were retained for 10 minutes	3 days, 6 days, 12 days	IL-2 in prostate↓ T in In serum↓ activity of Na^+^-K^+^-ATPase↑	Improve prostate histopathology

Bingcheng Hu/Zhenwang Ma [29]	Male wistar rats; DPT vaccine (0.5 ml，ip) ＋ protein purification liquid (15 mg/ml，sc) ＋ CFA (sc); 45 days	Shenhua thunder fire moxibustion	CV4, CV3, BL32, BL34	Moxibustion is away from the skin 2–3 cm 10 min	Once every day 28 days	IL-1*β*, TNF-*α*↓ in prostate	Improve prostate histopathology

Shengjie Zhang/Jia Li [44]	SPF male SD rats;agar solution (2%, 0.1 ml);injected into the prostate	Acupoint catgut embedding	CV4 BL23	Once every week	4 weeks	IgG, Zn in prostate↑	The activity of rats is increased↑; the amount of drinking water is increased↑; the urine turbidity is decreased↓
